# Negative results positive for understanding the neural representation of valence across the brain

**DOI:** 10.1016/j.neuron.2025.08.024

**Published:** 2025-09-17

**Authors:** Sounak Mohanta, Kay M. Tye

**Affiliations:** 1 Salk Institute for Biological Studies, La Jolla, CA 92037, USA; 2 Howard Hughes Medical Institute, Salk Institute for Biological Studies, La Jolla, CA, USA

## Abstract

In this issue of Neuron, Biane et al.^1^ used cellular-resolution recordings in ventral CA1 (vCA1) that it encodes stimulus features and identity, but not a generalized abstraction of valence. This suggests a complementary role of vCA1 to valence coding regions by providing stimulus-specific representations.

We are constantly bombarded by a battery of sensory stimuli, and have evolved to avoid things that harm us and approach things that help us. Valence processing may be ***the*** function best conserved across evolution – thus a fundamental question in neuroscience is:

How does our brain determine what is “good” or “bad”?

This mysterious procedure for rapidly detecting, filtering, evaluating and responding to sensory stimuli appropriately by avoiding threats and approaching rewards is known as valence processing^2^. A function this essential for survival has evolved many systems for redundancy and different levels of processing that can serve unique functions for quick responses, long-term memories, and dynamic valuations in a changing environment. Some stimuli require step-by-step processing to guide complex decisions and planning. Others demand immediate action. While a predator can be detected at a non-threatening distance with auditory or visual input, often tastes and somatosensory input imply proximal threat imminence. We posit that all of these levels of processing (fast, slow, dynamic) contribute to support optimal valence processing.

A decade ago, the question was: ***how does a single neuron qualify as encoding valence?***

When considering valence processing at the level of a ***single cell***, it must minimally meet the following criteria ^2,3^:

1. It must show phasic responses to sensory stimuli that differentiate between positive and negative valence (e.g. statistically distinguishable responses to sucrose and quinine)
2. This must be separable from simply coding stimulus features such as sensory modality (e.g. difference between response to sucrose and female odor is the same as the difference between sucrose and predator odor)

While this operational definition for what features a single neuron must possess to qualify as valence coding may be useful, it may be too myopic for understanding how valence is processed across distributed circuits.

Perhaps now, it is time to start asking: ***how does a complex system encode valence?***

When considering valence processing in a complex system, we must consider how function may make multiple demands on the form. Specifically, we may need some systems for rapid responses, for example in the case of imminent predatory threat. We may need other systems for long-term memory formation and generalization to create adaptive heuristics. For example, after being attacked by a lion, we may form a long-term memory that is generalized to a heuristic such that if we encounter a tiger, we may be better prepared to avoid the threat. Finally, we need systems for devaluation and revaluation, for example if we used to love ice cream but have since become lactose intolerant, we need to reassign valence incorporating the new memory to adjust the motivational significance of ice cream. Each of these functions are components of valence processing, and each is likely to be mediated by distinct circuitry. However, recently the field may have some bias towards finding “valence coding” neurons, unduly overlooking important other motifs that allow us to process valence with all the versatility of a complex organism.

In this issue of Neuron, Biane and colleagues^1^ perform a rigorous, comprehensive, and elegant investigation into whether CA1 neurons encode valence. Performing endoscopic calcium imaging using a genetically-encodable calcium indicator in vCA1 in conjunction with 6 classic valence experiments, they convincingly demonstrate that vCA1 does ***not*** encode valence as a compressed, abstract representation of motivational significance – instead it carries stimulus-specific representations.

Biane and colleagues conducted 6 experiments in vCA1 spanning 3 classic valence-probing paradigms: 1) comparison of stimuli with distinct sensory features to see if there is valence generalization, 2) association of valence to conditioned stimuli of matched modality predictive of outcome to see if neural representation of valence is transferred to conditioned stimuli, 3) devaluation of an unconditioned stimulus with conditioned taste aversion to see if encoding changes with value.

The authors did not see a proportion of neurons that decoded valence above chance, despite their thorough investigation. These results support the notion that valence generalization is unlikely to be a ubiquitous motif, given that reducing all the high-dimensional inputs down to a single dimension clarifies the robustness of valence coding, but reduces the speed, fidelity, and contextual specificity. Positively valenced stimuli may have distinct motor responses (female odor versus sucrose), and even bitter and sweet tastes evoke different motor responses. Another advantage of representing stimuli separately is to enable individual stimulus to be devalued or revalued in response to changing environment conditions and internal needs. The authors come to a decidedly negative conclusion about the existence of valence coding neurons within vCA1, but clarify the role of vCA1 in a “labeled lines” motif^2^ for valence processing (Figure 1).

In fact, the finding that vCA1 does not encode valence is consistent with its role in pattern completion/separation - a function that would ***not*** benefit from valence coding (because abstract generalization and stimulus discrimination require opposing computations). This distinction between brain regions that have more “compact” coding regimes like the amygdala, or “distributed” coding strategies like the cortex can be contrasted with the 3rd part of the so-called emotional triad, the vCA1. The work of Biane and colleagues highlights that vCA1 uses a structured coding regime that preserves stimulus features.This manuscript comes at an exciting moment in the history of valence processing research. The technology-induced explosion in neural circuits research in the past 20 years enabled us to survey the abundance of circuits in the brain that can produce approach or avoidance behaviors. However, as the field has matured, many circuits sufficient to promote approach or avoidance still lack neural dynamics that locally encode valence. For example a recent study in the cortical amygdala showed robust optogenetic effects on approach and avoidance, yet an absence of coding that was distilled down to valence^4^. In the cortical amygdala, this means that different odors that require distinct behavioral responses are represented separately, instead linking sensory inputs directly to motor responses.

It is likely that the majority of neural real estate devoted to valence processing performs valence coding in a manner that is a more direct line from stimulus to response – a simpler computation than distilling down to the positive or negative valence dimension (Figure 1).

Here, we argue that while the bar for a *single neuron* to be “valence coding” is rather restrictive and we would not predict that this functional cell type be common in the brain, as redundancy in coding strategies is important for a function so essential. At the same time, while single neurons in vCA1 do not autonomously encode valence, they are still involved in the expression of anxiety and fear behaviors^5^, and Biane and colleagues offer an important piece of the puzzle with their comprehensive recordings across multiple experiments investigating where sensory features are preserved in the representation versus those where they are abstracted away. Understanding what computations are performed within each biological substrate would allow us to truly understand how the brain determines what is good or bad for its survival, and all representational strategies likely serve a purpose.

## Figures and Tables

**Figure 1:**
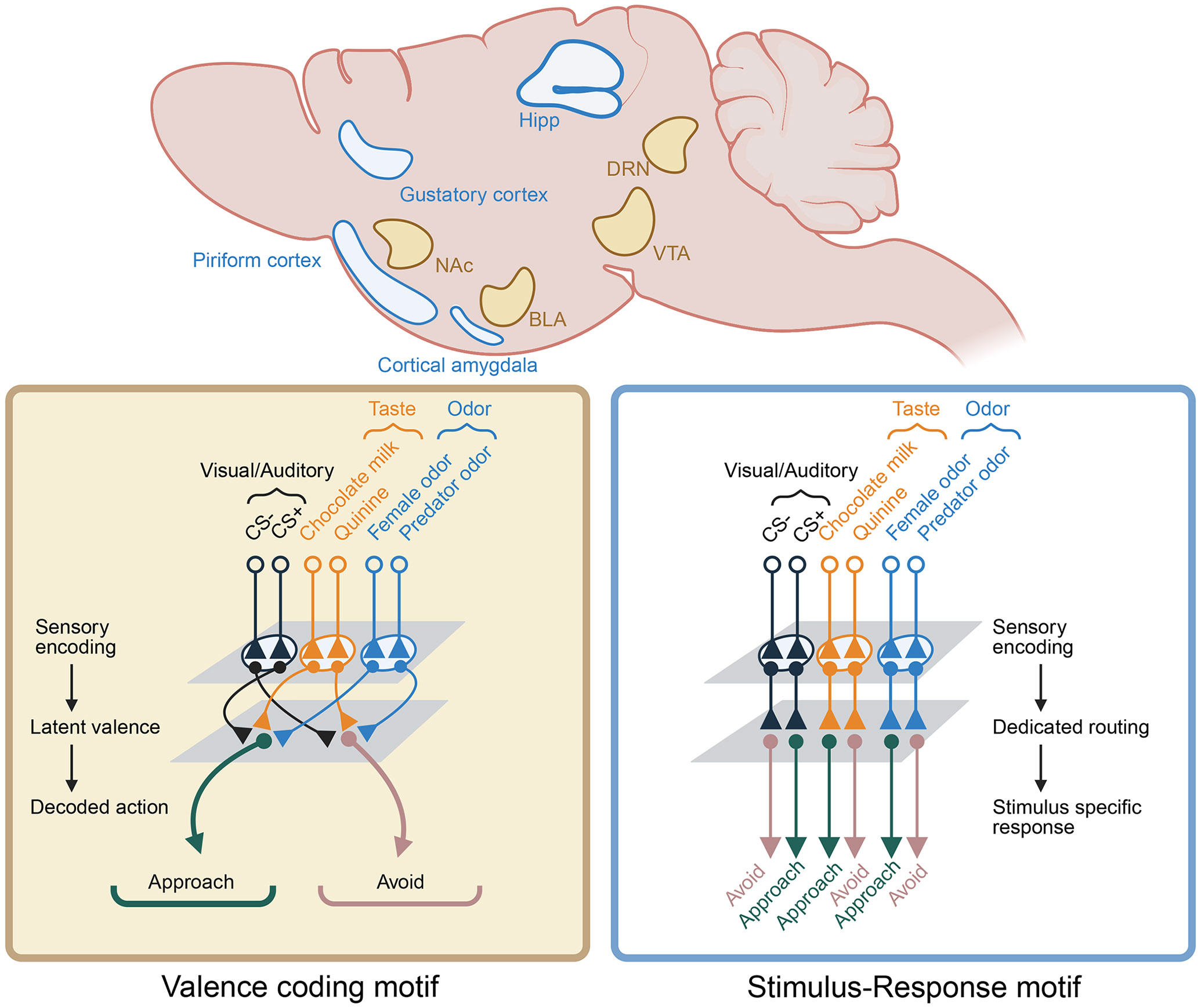
Neural motifs for encoding stimulus valence and guiding behavioral action. **Top:** Schematic of brain regions implicated in valence processing or supporting motivated behavior, including the nucleus accumbens (NAc), ventral tegmental area (VTA), dorsal raphe nucleus (DRN), hippocampus (Hipp), piriform cortex, cortical amygdala, gustatory cortex and basolateral amygdala (BLA). Color shading reflects existing evidence for distinct computational motifs: brown outlines indicate regions exhibiting and Valence Coding motif, while blue denotes regions favoring a Stimulus-Response motif. **Bottom left:** *Valence Coding motif*. In a valence coding motif, diverse input stimuli from different sensory modalities (e.g. visual, taste, odor etc.) are projected into a common latent space where the representation of stimuli is abstracted to positive and negative valence, irrespective of sensory modality or specific motoric output. This integrated representation allows for generalized, value-based decoding that drives behavioral responses such as approach or avoidance. Stimuli with similar motivational valence (e.g., chocolate milk and female odor) are grouped together in this space despite differing sensory features. Brain microcircuits implicated in this motif can be found in brain regions such as the BLA, NAc, VTA, DRN^3,6-8^. **Bottom right:** *Stimulus-Response motif*. In a stimulus-response motif, a “Labeled Lines” circuit architecture provides the advantage of speed and fidelity. Each stimulus is processed through dedicated, parallel channels with minimal convergence. This hard-wired architecture supports direct stimulus-to-action mapping (e.g., quinine → avoid, chocolate milk → approach) without reliance on a shared valence representation. Regions associated with this motif include the piriform cortex^9^, hippocampus^1^, and gustatory cortex^10^.
